# Validity, reliability and interpretability of the Thai version of the urticaria control test (UCT)

**DOI:** 10.1186/s12955-016-0466-y

**Published:** 2016-04-14

**Authors:** Kanokvalai Kulthanan, Leena Chularojanamontri, Papapit Tuchinda, Chuda Rujitharanawong, Marcus Maurer, Karsten Weller

**Affiliations:** Department of Dermatology, Faculty of Medicine, Siriraj Hospital Mahidol University, 2 Wanglang Road, Bangkok, 10700 Thailand; Department of Dermatology and Allergy, Allergie-Centrum-Charité, Charité-Universitätsmedizin Berlin, Berlin, Germany

**Keywords:** Chronic urticaria, Urticaria Control Test (UCT), Minimal clinically important difference (MCID), Reliability, Validity

## Abstract

**Background:**

The Long Form and Short Form of the German (original) version of the Urticaria Control Test (UCT) have shown to be valid and reliable instruments for assessing patients with all types of chronic urticaria (CU). The cutoff scores for identifying patients with well-controlled disease were ≥ 24 and ≥ 12 for Long and Short Forms, respectively. However, the sensitivity to change and minimal clinically important difference (MCID) of the UCT have never been systematically evaluated. This study aimed to investigate the validity, reliability, screening accuracy, sensitivity to change and MCID of the linguistically validated translation of the UCT into the Thai language for assessing CU in the Thai population.

**Methods:**

A structured translation and pre-testing were done to cross-culturally adapt the UCT for the Thai language. All measurement properties of both forms of the Thai UCT were validated in 169 patients with CU.

**Results:**

There were strong correlations between the Thai UCT score and disease activity, health-related quality of life impairment, and disease control (all correlations ≥ 0.7). Good internal consistency and excellent intra-rater reliability were demonstrated. The same cutoff scores to define patients with well-controlled disease should be used as those recommended for the original UCT version. MCIDs equated to increase in scores of 6 and 3 for the Long and Short Forms, respectively, of the Thai UCT should be used to identify patients who had minimal responses. Score increments of ≥10 and ≥ 6 for Long and Short Forms, respectively, should be used to define patients who had marked responses.

**Conclusions:**

This study confirmed the applicability of the UCT for use in Thailand, a country that has a very different language and cultural setting than that of Germany and the United States. Further studies are required to examine the suitability of the UCT for use in the pediatric population.

## Background

Chronic urticaria (CU) is a common debilitating skin disease which is characterized by the recurrence of wheals, angioedema, or both for longer than 6 weeks. Its symptoms fluctuate considerably from day to day making it difficult to assess disease activity and treatment response. Furthermore, it can significantly affect the patients’ health-related quality of life (HRQoL). To follow and treat patients with CU effectively, it is important to have valid and reliable tools to capture different dimensions of disease impact. Over the past decade, several attempts have been made to create specific instruments that can be used to evaluate patients with CU globally by jointly assessing disease activity, HRQoL impairment, and the use of symptomatic medications [1]. Three specific questionnaires for CU patients have been developed including the Urticaria Activity Score (UAS), the Chronic Urticaria Quality of Life Questionnaire (CU-Q_2_oL) and the Urticaria Severity Score [1–4]. Although the European Academy of Allergology and Clinical Immunology, Global Allergy and Asthma European Network, European Dermatology Forum and World Allergy Organization (EAACI/GA^2^LEN/EDF/WAO) guideline recommends the UAS and CU-Q_2_oL as the gold standard measurements for evaluating patients with CU in 2013 [5], both of them still have some limitations: (i) they are designed only for patients with chronic spontaneous urticaria, and (ii) they are not able to use to evaluate disease severity and impact of disease on patients’ HRQoL simultaneously [1]. In addition, clear cutoff values for the interpretation of their scores are missing.

The Urticaria Control Test (UCT) was devised and published in 2014 to overcome these limitations. It was originally developed in the German language and aimed to assess disease control in all types of CU patients [6]. Two forms of the UCT are available; the Long Form UCT (8 questions) and the Short Form UCT (4 questions). Since the results of both UCT forms have been found to correlate extensively, the Short Form UCT is sufficient for most settings and primarily recommended for clinical trials and routine patient care [6]. Each UCT question has five answer options (0–4 points) regarding to the disease activity during the past four weeks. The maximum scores of Long and Short Forms are 32 and 16, respectively. The lower the score is, the lower the disease control and the higher the disease activity [6]. Previous study has shown that both forms of the original UCT are valid and reliable instruments for the management of patients with CU in clinical practice. The cutoff scores for identifying patients with well-controlled disease were ≥ 24 and ≥ 12 for Long and Short Forms, respectively [6]. However, the minimal clinically important difference (MICD), which is the smallest difference in score that patients perceive as noticeable improvement, has never been evaluated. The current study aimed to investigate the validity, reliability, screening accuracy, sensitivity to change and MCIDs of the linguistically validated Long Form and Short Form of the newly developed Thai version of the UCT.

## Methods

### Translation of the long and short forms of the UCT questionnaires

The original (German) version of the UCT was independently translated into Thai language by two native Thai speakers with a command of German language. Then, these two Thai versions were reviewed for comprehensibility by dermatologists specialized in allergy. After these dermatologists reached consensus, the final Thai version was back-translated into German by two independent bilingual translators. The back-translated versions were then reviewed against the originals by the original authors. Potential misconceptions or misinterpretations introduced in the translation process were discussed between Thai research team and the original authors. After a consensus on final Thai language version was achieved, the Thai version of UCT was tested in 10 CU patients (cognitive debriefing interviews). Here, no points of misunderstanding were detected. Subsequently, the final Thai version of UCT was administered for the study.

### Clinical measures

(i.) *Urticaria Activity Score 28* (*UAS28*): The UAS28 is a prospective diary-type patient-reported outcome (PRO) measure to assess disease activity of CU patients for four weeks. It sums up the number of wheals and the intensity of pruritus on a four-point scale (0–3) with a minimum and maximum score of 0 and 6 points per day, respectively. The UAS28 scores range from 0–168 [2].
(ii.) *The validated Thai version of the Chronic Urticaria Quality of Life Questionnaire* (*CU*-*Q*_*2*_*oL*): The CU-Q_2_oL comprises 23 items categorized into six domains: pruritus (two items), impact on daily activities (six items), sleep problems (five items), limitations (three items), look (five items), and swelling (two). For each item, patients were asked to choose between five response options (scored 1–5) indicating the intensity of each item in the last two weeks. A total score across all items was calculated and transformed into scores ranging from 0 to 100, with a score of 100 indicating the worst possible HRQoL impairment. The original authors of CU-Q_2_oL kindly gave formal permission to Dr. Kulthanan to validate and use the Thai version of CU-Q_2_oL [3, 7].
(iii.) *Physician*’*s global assessment of disease control*: The Physician’s global assessment-visual analog scale (PhyGA-VAS) is a physician evaluation instrument for assessing disease control during the last four weeks. It is a 10-cm unmarked line which ranges between 0 cm (not at all under control) and 10 cm (completely under control) [6].
(iv.) *Patient*’*s global assessment of disease severity*: the patient’s global assessment-visual analogue scale (PatGA-VAS) was used to assess disease severity during the previous four weeks. The PatGA-VAS is an unmarked line anchored at the two ends with “no complaints” (0 cm) and “maximal complaints” (10 cm) [6].
(v.) *Patient*’*s global assessment of disease control*. Patient’s global assessment-Likert scale (PatGA-LS) was used to assess treatment sufficiency during the previous four weeks. The PatGA-LS is a 5-point scale for assessing disease control (0 = no control, 1 = little control, 2 = moderate control, 3 = good control, and 4 = complete control) [6].

### Subjects

Thai patients with CU aged 18 years or older attending Allergy Clinic, Department of Dermatology Siriraj Hospital were invited to participate and recruited into the study. Informed consent was obtained from all individual participants included in the study. Patients who were not able to read Thai and had other dermatologic and mental diseases were excluded. All measurement properties of the validated Thai UCT were assessed in 168 CU patients. This study (NCT 02285049) was approved by Siriraj Institutional Review Board, Faculty of Medicine Siriraj Hospital. The ethics approval number is SI 558/2014.

On the first visit (day 0), patients were instructed how to complete the questionnaires: (i) the validated Thai version of CU-Q_2_oL, (ii) the validated Thai version of UCT, (iii) UAS28, (iv) PatGA-VAS, and (iv) PatGA-LS. After patients understood how to complete the questionnaires, they would be asked to complete the UAS28 by themselves prior to the second visit. At the second visit (day 28), the UAS28 was collected and the validated Thai version of UCT and CU-Q_2_oL, PatGA-VAS and PatGA-LS were completed by the patients at Allergy Clinic, Department of Dermatolgy, Siriraj Hospital. All PhyGA-VAS forms were completed by one physician. Another UAS28 was given to each patient to complete for 28 consecutive days before coming back to the hospital on the third visit. The same process was repeated on the third visit (day 56). During each visit, the participants received appropriate treatment according to their disease severity and the EAACI/GA^2^LEN/EDF/WAO guideline [5].

### Statistical analysis

The methodological quality and statistical analysis of this study was based on Consensus-based Standards for the selection of health Measurement INstruments (COSMIN) and the Statistical Package for the Social Sciences (SPSS, Inc., Chicago, IL, USA) version 18 [8].

1. Validity
  - ***Construct validity*** measures the degree to which a relevant construct is measured. The correlation between the validated Thai version of UCT and CU-Q_2_oL, UAS28, PhyGA-VAS, PatGA-VAS, and PatGA-LS were evaluated by Pearson’s correlation coefficient. Weak, moderate and strong correlations were defined as correlation coefficient values of < 0.3, 0.3-0.6, and > 0.6 respectively [9].
  - ***Known***-***groups validity*** measures the capacity of discrimination across groups that are assumed to differ. The capacity of the Thai UCT to differentiate patients with different levels of urticaria severity (UAS28) was explored using Kruskal Wallis test. The UAS28 scores of ≤10, 11–35, 36–70, 71–105 and ≥106 were used to classify the disease activity of the patients into none, mild, moderate, severe and very severe, respectively [6].
2. Reliability
  - ***Internal consistency*** measures the correlations among items of measurement. The internal consistency was determined by Cronbach’s α reliability coefficient. Excellent, good and acceptable reliability were defined as α ≥ 0.9, 0.7 ≤ α < 0.9, and 0.6 ≤ α < 0.7, respectively [10].
  - ***Test***-***retest reliability*** measures the consistency of the UCT across multiple administrations. Stable patients (no change in disease control by PatGA-LS score during 4-week interval) should display comparable UCT scores at two different administrations (2nd and 3rd visits). Intraclass correlation coefficient (ICC) values of <0.40, 0.40-0.75, and >0.75 were regarded to indicate poor, average, and strong reliability, respectively [11].
3. Screening accuracy
  - ***Screening accuracy*** (***categorization***) is the ability of UCT to categorize patients into suffering from poorly-controlled and well-controlled disease. Patients who had PatGA-LS scores of 0, 1, and 2 were defined as having poorly-controlled urticaria while patients who had PatGA-LS scores of 3 and 4 were defined as well-controlled. Receiver operating characteristic (ROC) and area under the curve (AUC) were used to analyze screening accuracy. AUCs of 1, 0.9, 0.8, 0.7, and 0.5 were defined as perfect, excellent, good, fair, and no better than chance, respectively [12].
4. Sensitivity to change and Interpretability (MCID)
  - ***Sensitivity to change*** is the ability of the UCT to detect change over time in the construct to be measured. As PatGA-LS is a global rating scale assessing disease control, we expect a positive correlation between changes in the PatGA-LS and changes in the Thai UCT score. ROC analysis and AUC were used to determine the sensitivity to change of the Thai UCT. The larger the AUC is, the better the ability of the Thai UCT to detect change [12].
  - ***Interpretability*** measures the capacity of a questionnaire to be interpreted from quantitative scores or change in scores to a qualitative meaning. The MCID is the minimal difference in a score that patients recognized as a meaningful improvement [13, 14]. Three different methods were applied to investigate the MCIDs of the validated Thai versions of the UCT.

(i) Distributional methods look at the statistical distribution of the instruments values. The standard error of measurement (SEM) and one-half of standard deviation (SD) of the measure of interest are most widely accepted to represent MCID values. SEM was calculated using SD at baseline of UCT score × (1-reliability of the validated Thai version of UCT) ^1/2^. Distributional methods were used to derive MCID-1 [15–17].
(ii) ROC analyses determine the sensitivity and specificity over the range of the absolute reductions in the Thai UCT score of patients who were “minimal responders” and “marked responders” [18]. Two criteria: (i) an increase in PatGA-LS score = 1 and (ii) an increase in PatGA-LS score = 2 were used to define “minimal responders” and “marked responders”, respectively.
(iii) Anchor-based approaches compare changes in an instrument’s score with an “anchor” as a reference [18]. We wished to investigate: (i) the difference in the mean scores of the Thai UCT between “minimal responders (an increase in PGA score = 1)” and “non-responders (no change in PGA score)” and (ii) the difference in the mean scores of Thai UCT score between “marked responders (an increase in PGA score = 2)” and “non-responders (no change in PGA score and an increase in PGA score = 1)”.

## Results

**Fig. 1:**
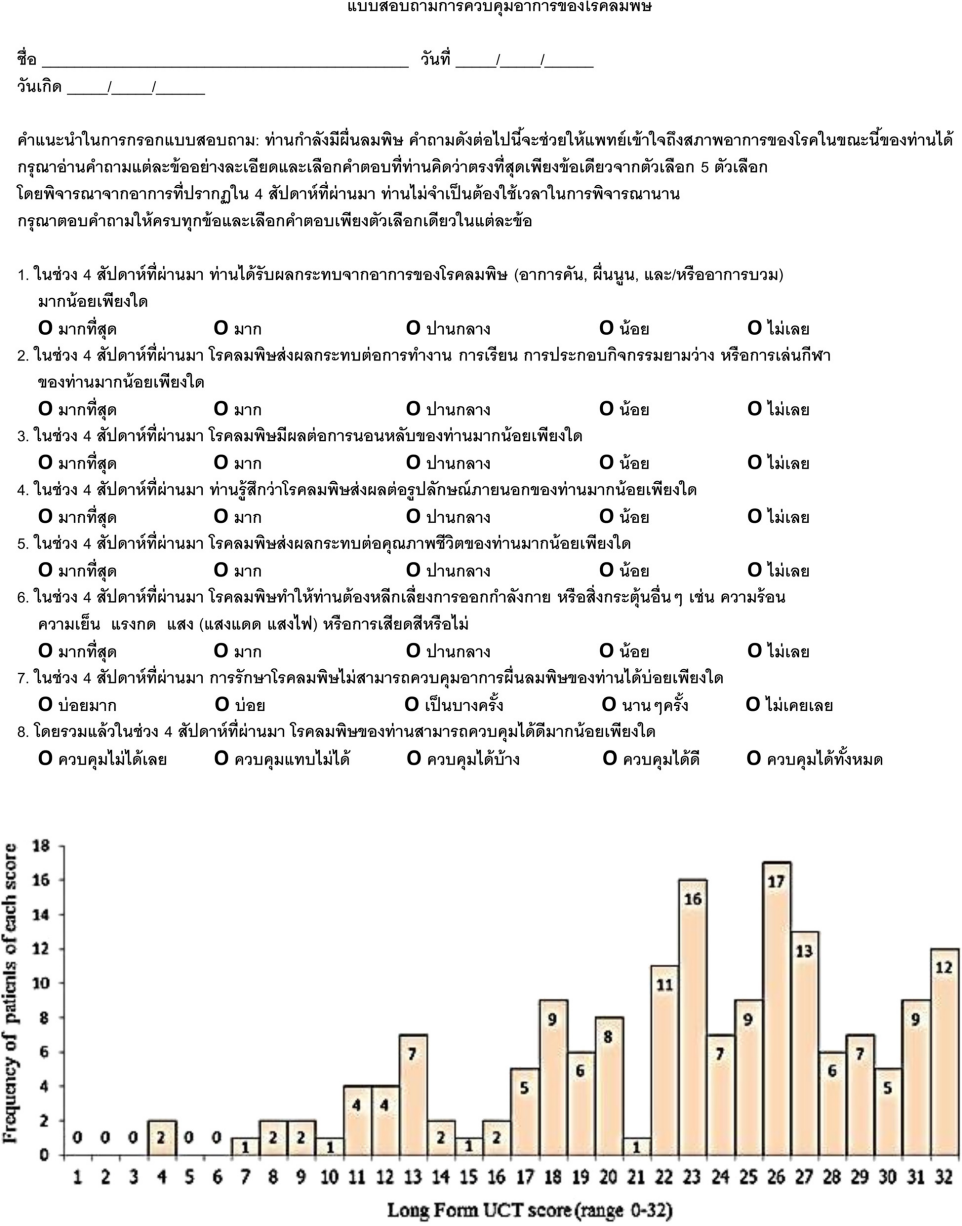
Distribution of scores of the Long Form of the validated Thai Urticaria Control Test

**Fig. 2:**
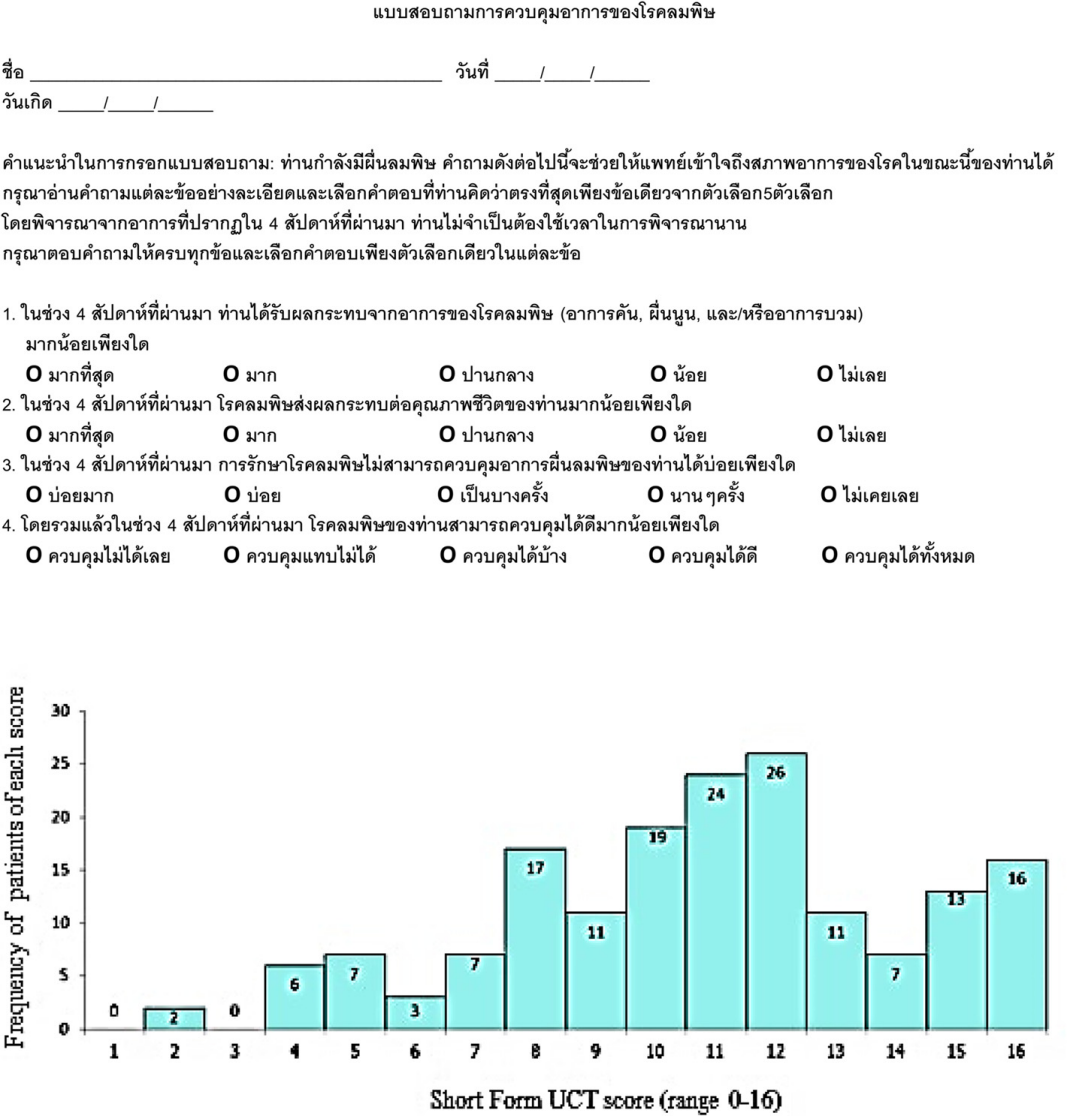
Distribution of scores of the Short Form of the validated Thai Urticaria Control Test

Of 169 patients with CU (mean ± SD age, 42 ± 13.9 years), 132 (78.1 %) patients were female. Chronic spontaneous urticaria was the predominant diagnosis (98 %). The average duration of disease was two years ranging from two months to 34 years. The mean ± SD UCT score at baseline for Long and Short Forms of Thai UCT were 22.72 ± 6.57 and 10.79 ± 3.36, respectively. A wide distribution of scores was obtained for each form (Figs. 1 and 2).

### Construct and known-groups validity

Tables 1 and 2 demonstrates that both forms of the Thai UCT showed a strong correlation with disease activity/severity (UAS28, PatGA-VAS), disease control (PatGA-LS, PhyGA-VAS) and HRQ_2_oL impairment (the validated Thai version of CU-Q_2_oL). There were statistically significant differences in UCT scores of patients with different levels of disease severity (*p* < 0.0001).

**Table 1:** Construct and Known-groups Validity of the Thai-version of the Urticaria Control Test (UCT)

Construct validity (correlations)	Urticaria Control Test	
	Long Form	Short Form
Urticaria Activity Score (UAS28)	−0.67*	−0.68*
Patient’s global assessment of disease severity (PatGA-VAS)	−0.81*	−0.72*
Patient’s global assessment disease control (PatGA-LS)	0.76*	0.83*
Physician’s global assessment of disease control (PhyGA-VAS)	0.71*	0.77*
Validated Thai version of Chronic Urticaria Quality of Life Questionnaire (CU-Q_2_oL)	−0.89*	−0.78*

*p value = < 0.0001, *r* > 0.60 (strong correlation). Please note that some correlations show negative values as higher scores of the UCT (well controlled disease) indicates lower disease severity (UAS, PatGA-VAS) and negative impact on healh-related quality of life (CU-Q_2_oL)

**Table 2:** Construct and Known-groups Validity of the Thai-version of the Urticaria Control Test (UCT)

Known-groups validity	Urticaria Control Test					
	Long Form			Short Form		
Urticaria Activity Score (UAS28)	*n of patients*	*Mean* (*SD*)	*Median*	*n of patients*	*Mean* (*SD*)	*Median*
UAS28 ≤ 10	55	29.1 (3.3)	30.0	55	14.1 (2.0)	15.0
UAS28 = 11-35	61	24.5 (4.7)	25.0	61	11.5 (2.4)	12.0
UAS28 = 36-70	31	20.0 (5.8)	21.0	31	8.7 (2.8)	9.0
UAS28 = 71-105	14	19.9 (5.0)	20.0	14	8.8 (2.2)	8.0
UAS28 ≥ 106	8	13.5 (*6.9*)	14.0	8	5.8 (3.1)	5.0

Please note that some correlations show negative values as higher scores of the UCT (well controlled disease) indicates lower disease severity (UAS, PatGA-VAS) and negative impact on healh-related quality of life (CU-Q_2_oL)

### Internal consistency and test-retest reliability

The Cronbach’s alpha values of the Long and Short Forms of Thai UCT were 0.91 and 0.86, respectively which indicates excellent internal consistency. Thirty-nine patients who had no change in PatGA-LS scores during the four-week interval between the 2nd and the 3rd visit were included to analyze for test-retest reliability. The ICCs of the Long and Short Forms of Thai UCT were 0.98 (95 % confidence interval = 0.96-0.99) and 0.99 (95 % confidence interval = 0.97-0.99) which demonstrated strong intra-rater reliability of Thai UCT.

### Screening accuracy (Categorization)

Using PatGA-LS, 100 and 94 patients were classified as having well-controlled disease at 2nd and 3rd visits, respectively. The AUCs on ROC analyses demonstrated excellent accuracy of both forms of Thai UCT to categorize patients into having poorly-controlled and well-controlled disease (Table 3). For the Long Form UCT, UCT scores of ≥ 24 (sensitivity 84.7 %, specificity 78.7 %) or ≥ 25 (sensitivity 79.6 %, specificity 87.2 %) were found to be suitable cutoff values to define well-controlled disease. For the Short Form UCT, UCT scores of ≥ 11 (sensitivity 90.8 %, specificity 80.9 %) or ≥ 12 (sensitivity 79.6 %, specificity 93.6 %) were suitable cutoff values to define well-controlled disease.

**Table 3:** Cutoff values for the UCT to screening patients for well and poorly controlled disease

Thai UCT score (Long Form)	Patient’s global assessment of disease control (PatGA-LS)			
	2nd visit		3rd visit	
	Well-controlled patients (*n* = 100)		Well-controlled patients (*n* = 94)	
	Sensitivity (%)	Specificity (%)	Sensitivity (%)	Specificity (%)
20.0	75.2	81.3	92.9	44.7
21.0	77.0	76.8	92.9	53.2
22.0	76.8	75.4	89.8	59.6
23.0	81.2	73.5	87.8	68.1
24.0	**90.6**	**72.6**	**84.7**	**78.7**
25.0	**94.6**	**71.4**	**79.6**	**87.2**
26.0	95.7	66.0	73.5	93.6
AUC	0.89 (0.84-0.94)		0.90 (0.85-0.95)	
Thai UCT score (Short Form)	Patient’s global assessment disease control (PatGA-LS)			
	2nd visit		3rd visit	
	Well-controlled patients (*n* = 100)		Well-controlled patients (*n* = 94)	
	Sensitivity (%)	Specificity (%)	Sensitivity (%)	Specificity (%)
9.0	67.6	93.9	95.9	48.9
10.0	72.6	91.1	93.9	63.8
11.0	**76.6**	**84.5**	**90.8**	**80.9**
12.0	**92.0**	**84.0**	**79.6**	**93.6**
13.0	97.0	70.9	61.2	97.9
AUC (95 % CI)	0.93 (0.89-0.97)		0.93 (0.89-0.97)	

Abbreviations: *AUC* (95 % CI): area under the curve (95 % confidence interval)

Bold entries indicate the most important results

### Sensitivity to change and interpretability (MCID)

The correlations between change in PatGA-LS and Long Form UCT and between change in PatGA-LS and Short Form UCT were 0.60 and 0.70, respectively. These strong correlations indicated that the PatGA-LS can be used as a construct approach of sensitivity to change and MCID. The MCID-1 values for the Long Form UCT were 3.9 (0.5 SD), 2.0 (SEM) using Cronbach’s α value, and 0.9 (SEM) using ICC value. For Short Form, the MCID-1 values were 1.7 (0.5 SD), 1.2 (SEM) using Cronbach’s α value, and 0.3 (SEM) using ICC value. Table 4 demonstrated a good sensitivity of the Thai UCT to detect change over time (all AUC ≥ 0.8). On ROC analyses, the smallest mean changes that identified “minimal responders” for the Long Form and Short Form were 3 and 2, respectively. The smallest mean changes that identified “marked responders” for Long and Short Forms were 4 and 3, respectively (Table 4). Table 5 showed that the differences of mean scores between “minimal responders” and non-responders” were 5.9 and 2.8 for Long and Short Forms, respectively. The differences of mean scores between “marked responders” and “non-responders” were 9.6 and 5.8 for Long and Short Forms, respectively. Therefore, the three different approaches demonstrated that MCIDs that identified “minimal responders” and “marked responders”, respectively for the Long Form UCT ranged from 0.9-5.9 and 0.9-9.6. On the other hand, the MCIDs that identified “minimal responders” and “marked responders” for the Short Form UCT were 0.3-2.8 and 0.3-5.8, respectively.

**Table 4:** The MCID by Receiver operating characteristic analysis

Changes in Long Form Thai UCT score	Patient’s global assessment of disease control (PatGA-LS) score			
	Change in score of 1 (minimal responders = 20)		Change in score of 2 (marked responders = 9)	
	Sensitivity (%)	Specificity (%)	Sensitivity (%)	Specificity (%)
1.0	90.0	74.1	100	64.7
2.0	90.0	81.9	100	71.3
3.0	85.0	85.3	88.9	75.0
4.0	70.0	90.5	88.9	81.6
5.0	60.0	94.8	77.8	86.8
6.0	45.0	97.4	77.8	91.2
AUC (95 % CI)	0.86 (0.80-0.98)		0.92 (0.86-0.99)	
Changes in Short Form Thai UCT score	Patient’s global assessment of disease control (PatGA-LS) score			
	Change in score of 1 (minimal responders = 20)		Change in score of 2 (marked responders = 9)	
	Sensitivity (%)	Specificity (%)	Sensitivity (%)	Specificity (%)
1.0	80.0	79.3	100	70.6
2.0	75.0	88.8	100	79.4
3.0	60.0	95.7	88.9	87.5
4.0	40.0	97.4	77.8	91.9
5.0	20.0	99.1	77.8	96.3
AUC (95 % CI)	0.84 (0.80-0.97)		0.96 (0.92-0.99)	

Abbreviations: *MCID* minimal clinically important difference, *AUC* (95 % CI): area under the curve (95 % confidence interval)

**Table 5:** The differences of mean scores of Thai UCT between minimal and non-responders and between marked and non-responders

Long Form of the Thai UCT					
Total mean score (SD.)		Between minimal responders and non-responders		Between marked responders and non-responders	
Baseline (*n* = 169)	Week 4 (*n* = 169)	Mean score (SD.) of minimal responders (*n* = 20)	Mean score (SD.) of non-responders (*n* = 116)	Mean score (SD.) of marked responders (*n* = 9)	Mean score (SD.) of non-responders (*n* = 136)
22.72 (6.57)	24.27 (6.27)	6.5 (7.3)	0.6 (2.7)	11.1 (7.2)	1.5 (2.7)
The differences of mean scores		5.9		9.6	
Short Form of the Thai UCT					
Total mean score (SD.)		Between minimal responders and non-responders		Between marked responders and non-responders	
Baseline (*n* = 169)	Week 4 (*n* = 169)	Mean score (SD.) of minimal responders (*n* = 20)	Mean score (SD.) of non-responders (*n* = 116)	Mean score of marked responders (*n* = 9)	Mean score of non-responders (*n* = 136)
10.79 (3.31)	11.31 (3.36)	3.0 (2.7)	0.2 (1.3)	6.3 (2.7)	0.6 (1.9)
The differences of mean scores		2.8		5.8	

Abbreviation: *SD* standard deviation

## Discussion and conclusions

The current urticaria guideline clearly recommends that the aim of treatment should be to achieve complete control of symptoms. However, none of the previously available patient reported outcome tools are designed to detect disease control in patients with CU [5]. This fact was one major reason why the UCT has been developed. In addition, the UCT was designed to be (i) a patient-reported outcome (PRO), (ii) independent of any previous patient presentations, (iii) easy to administer and fast to complete, and (iv) easy to score and interpret [6]. The validity, reliability, screening accuracy and feasibility of the original version has been proved for the original German version [6]. The reduction of the Long Form to the Short Form was not found to affect the performance of the UCT. Thus, the authors of the German version recommend to use the Short Form in routine clinical practice and the Long Form in case of additional information is required [6]. The German versions were also translated and linguistically validated for American English [6].

Our study demonstrated positive strong correlations of both forms of the Thai UCT with PatGA-LS and PhyGA-VAS. Notably, both forms of the Thai UCT correlated more strongly with PatGA-LS than PhyGA-VAS, probably because PatGA-LS was a self-assessment of the patients as is the UCT. In addition, strong negative correlations were found between the Thai UCT and UAS28, PatGA-VAS, and the validated version of Thai CU-Q_2_oL because higher scores of the UCT (well-controlled disease) indicates lower disease severity and lower negative impact on HRQoL of patients. Cronbach’s α and ICC values of the Thai and German versions of the UCT were comparable which indicated excellent reproducibility of both versions of the UCT [6, 10, 11]. For screening accuracy, the cutoff values we obtained were very close to those of the original study. In order to facilitate the use of UCT, we propose that the same cutoff values for well controlled- and poorly controlled disease of ≥ 24 and < 24 for Long Form UCT and ≥ 12 and < 12 for Short Form UCT, respectively should be used for the Thai version as those recommended in the original German version [6].

The sum of weighted scores for the use of antihistamines, oral glucocorticoids, ciclosporin, hydroxychloroquine and montelukast has been used to determine disease severity and treatment outcome of CU patients in several studies. However, its sensitivity to change and MCID have never been defined [19–21]. Both sensitivity to change and MCID are important measurement properties that can be used to objectively determine treatment outcomes. Moreover, it is generally accepted that anchor-based methods have higher clinical relevance and should be preferred to define MCID rather than statistical distribution-based analysis. For practical use, we therefore propose that score increments of 6 and 3 for the Long and Short Forms of Thai UCT, respectively can be regarded as the smallest increases that identify “minimal responders” well. Scores increments of ≥10 and ≥ 6 for Long Form and Short Form Thai UCT, respectively may be used to identify “marked responders”.

## Conclusions

This study cleary confirms the usefulness of the UCT to assess disease control and to guide treatment decisions in patients with chronic urticaria in the Thai population, which can be considered quite different in terms of the cultural setting and the language spoken from the German and US-American populations the original UCT has been developed for. Moreover, this study adds important new information to help in the interpretation of UCT results. For the first time, sensitivity to change and interpretability have been confirmed and defined. We are convinced that both forms of the UCT are valid and reliable patient reported outcome instruments for use in routine clinical practice and clinical trials in European, North American and Asian countries. Since the UCT has been developed for adults, further studies are required to examine the UCT in children and adolescents.

## Abbreviations

AUC: area under the curve
COSMIN: consensus-based standards for the selection of health measurement instruments
CU: chronic urticaria
CU-Q_2_oL: chronic urticaria quality of life questionnaire
EAACI/GA^2^LEN/EDF/WAO: the European academy of allergology and clinical immunology, global allergy and asthma European network, European dermatology forum and world allergy organization
HRQoL: health-related quality of life
ICC: intraclass correlation coefficient
MCID: minimal clinically important difference
PatGA-LS: patient’s global assessment-Likert scale
PhyGA-VAS: physician’s global assessment-visual analog scale
PRO: patient-reported outcome
ROC: receiver operating characteristic
SD: standard deviation
SEM: standard error of measurement
UAS: urticaria activity score
UCT: urticaria control test
